# The impact of mobile health applications on the outcomes of patients with chronic kidney disease: a systematic review and meta-analysis

**DOI:** 10.25122/jml-2023-0153

**Published:** 2023-09

**Authors:** Muhammad Thesa Ghozali, Satibi Satibi, Gerhard Forthwengel

**Affiliations:** 1 Department of Pharmaceutical Management, School of Pharmacy, Faculty of Medicine and Health Sciences, Universitas Muhammadiyah Yogyakarta, Yogyakarta, Indonesia; 2 Department of Pharmaceutics, Faculty of Pharmacy, Universitas Gadjah Mada, Yogyakarta, Indonesia; 3 Fakultat III, Hochschule Hannover, University of Applied Sciences and Arts, Hannover, Germany

**Keywords:** chronic kidney disease, meta-analysis, mobile health apps, self-management, CKD: Chronic Kidney Disease, RCT: Randomized Controlled Trial, SBP: Systolic Blood Pressure, DBP: Diastolic Blood Pressure, NIH: National Institute of Health, SIGLE: System for Information on Grey Literature in Europe, VHL: Virtual Health Library, NYAM: New York Academy of Medicine, ICTRP: International Clinical Trials Registry Platform

## Abstract

Chronic kidney disease is one of the main causes of mortality worldwide. It affects more than 800 million patients globally, accounting for approximately 10% of the general population. The significant burden of the disease prompts healthcare systems to implement adequate preventive and therapeutic measures. This systematic review and meta-analysis aimed to provide a concise summary of the findings published in the existing body of research about the influence that mobile health technology has on the outcomes of patients with the disease. A comprehensive systematic literature review was conducted from inception until March 1^st^, 2023. This systematic review and meta-analysis included all clinical trials that compared the efficacy of mobile app-based educational programs to that of more conventional educational treatment for the patients. Eleven papers were included in the current analysis, representing 759 CKD patients. 381 patients were randomly assigned to use the mobile apps, while 378 individuals were assigned to the control group. The mean systolic blood pressure was considerably lower in the mobile app group (MD -4.86; 95%-9.60, -0.13; p=0.04). Meanwhile, the mean level of satisfaction among patients who used the mobile app was considerably greater (MD 0.75; 95% CI 0.03, 1.46; p=0.04). Additionally, the mean self-management scores in the mobile app groups were significantly higher (SMD 0.534; 95% CI 0.201, 0.867; p=0.002). Mobile health applications are potentially valuable interventions for patients. This technology improved the self-management of the disease, reducing the mean levels of systolic blood pressure with a high degree of patient satisfaction.

## INTRODUCTION

Chronic kidney disease, often known as CKD, is one of the main causes of mortality worldwide. It affects approximately 800 million patients globally, accounting for approximately 10% of the general population. The increase in the incidence of CKD can be linked to the rise in the prevalence of health conditions related to CKD, including being overweight or obese, getting older, developing diabetes, or having high blood pressure [1]. Additionally, end-stage renal disease caused by CKD requires dialysis treatment, which is extremely expensive. The financial burden of CKD costs the healthcare systems more than 3% of annual health expenditures. This burden is considerably more critical in developing countries due to additional poverty and poor infrastructures [2, 3]. The significant burden of CKD prompts healthcare systems to implement adequate preventive and therapeutic measures [4, 5]. Managing CKD is challenging, including early prevention, timely diagnosis, efficient therapy, and continuous monitoring [6]. Notably, CKD has an indolent course and is usually associated with multiple comorbidities and poor prognosis. In order to provide treatment for patients with CKD, a multidisciplinary team consisting of medical doctors, researchers, and engineers is required. Individuals who suffer from CKD have poor awareness and a restricted comprehension of the progression of the disease [7].

Mobile health technology is an evolving approach to caring for patients with chronic disorders. Tablets, cell phones, and web-based portals are all examples of mobile devices that can be used to access this technology. Mobile health technologies promote communication between patients and their healthcare professionals, making it easier for patients to monitor their health at home and allowing for earlier diagnosis of any deterioration in their condition [8, 9]. Healthcare mobile applications generating personal health records may decrease the problems related to primary healthcare at remote extinctions. Given the complexity of delivering effective healthcare services, patients with CKD will likely benefit from real-time, personalized, interactive mobile healthcare applications. However, developing these applications is complex, and choosing the most useful application can be overwhelming for patients [10, 11]. Accurate knowledge and monitoring of CKD have an essential role in self-management. Slowing the progression of CKD necessitates significant personal involvement. Patients face complex recommendations on lifestyle modification, adherence to medications, and nutritional guidelines [12]. Traditional educational methods remained limited, with a short-term increase in disease knowledge [13-15]. This brought to light the importance of developing innovative methods to slow down the advancement of the disease. There has been limited research in the field regarding the effectiveness of mobile health education in improving understanding and self-care among individuals with chronic kidney disease. Therefore, further research in this area is warranted. This is attributable to the insufficient number of well-structured clinical studies that evaluated these outcomes. This information could be useful for patients with CKD in various ways, including disease monitoring, interpreting the effects of at-home treatments, maintaining complex prescription regimens, and adhering to food and hydration recommendations [16]. Herein, the purpose of this review was to compile information from the published literature on how mHealth solutions affect CKD patients' awareness, management, and outcomes.

## MATERIAL AND METHODS

This meta-analysis was conducted in accordance with the PRISMA (Preferred Reporting Items for Systematic Reviews and Meta-Analyses) [17] and Cochrane [18] criteria (Supplementary Table 1).

Supplementary Table 1

### Data Source

A comprehensive systematic literature review was conducted from the beginning of the study until its completion on March 1, 2023, using the following databases: PubMed, Google Scholar, Web of Science (ISI), Scopus, System for Information on Grey Literature in Europe (SIGLE), Virtual Health Library (VHL), New York Academy of Medicine (NYAM), Clinical trials, Controlled Trials (mRCT), EMBASE, Cochrane Collaboration, and WHO International Clinical Trials Registry Platform (ICTRP). This review covered the period from the study. No limitations were placed on the patients regarding their age, gender, ethnicity, language, race, or location.

The search methodology employed controlled vocabulary terms within the parameters of the searched databases. Medical subject headings in conjunction with text words were employed to ensure that a comprehensive selection of articles was examined. A manual search was conducted, including all relevant references from the retrieved articles. The approach of cross-referencing was utilized until it was determined that there was no other pertinent research. The following phrases served as inspiration for each possible combination: “Chronic”, “Kidney”, “renal”, “CKD”, “Dialysis”, “Mobile”, “m-health”, “mhealth”, “smartphone”, “smartphone”, “tablet”.

### Eligibility criteria

This systematic review and meta-analysis included all clinical studies that compared the efficacy of educational programs delivered via mobile applications to conventional educational treatments for patients with chronic renal disease. We excluded research for which data extraction was not feasible, studies conducted on animals, reviews, case reports, guidelines, letters, editorials, posters, comments, or book chapters. Articles without a comparison group were disregarded. Two independent reviewers conducted the initial screenings, and any discrepancies were resolved through discussion. The PRISMA Flowchart illustrates the reasons for study exclusions and the screening processes.

### Data extraction

Information regarding the characteristics of the included studies, such as titles, authors, publication years, registration numbers, study designs, time frames, and locations, was extracted from the relevant articles. Patients' age, gender, race, marital status, level of education, annual income, and number of comorbidities were among the demographic information collected at baseline. Data related to CKD included stage, duration of illness, and results of renal function tests. In addition, data related to mobile applications were extracted, including the operating system, duration of sessions, type of mobile app use, and features. Variables associated with the outcomes of interest were also reviewed, including the burden of kidney disease, renal function test results, medication adherence, patient satisfaction, self-management of CKD, body weight, and blood pressure.

### Risk of bias and quality assessment

The risk of bias in the included randomized controlled trials (RCTs) was assessed using the methodology developed by the Cochrane Collaboration [19]. For observational studies, their quality was evaluated using a method established by the National Institutes of Health (NIH) [20].

### Statistical analysis

In studies involving continuous variables, researchers commonly employ either the weighted mean difference (WMD) or the standardized mean difference (SMD). To convert data originally presented as a median and range into a mean and standard deviation (SD), the formulas developed by Hozo *et al*. [21] were applied. When a consistent effect size was observed across the population, the fixed-effect model was used; otherwise, the random-effects model was applied. The statistical homogeneity of the data was determined by utilizing the Higgins I2 statistic, with a value of more than fifty percent, and the Cochrane Q (Chi2 test), with a value of less than ten percent [22]. Data analysis was performed using Review Manager, version 5.4 [23, 24], and significance was determined when the p-value was less than 0.05.

## RESULTS

**Figure 1:**
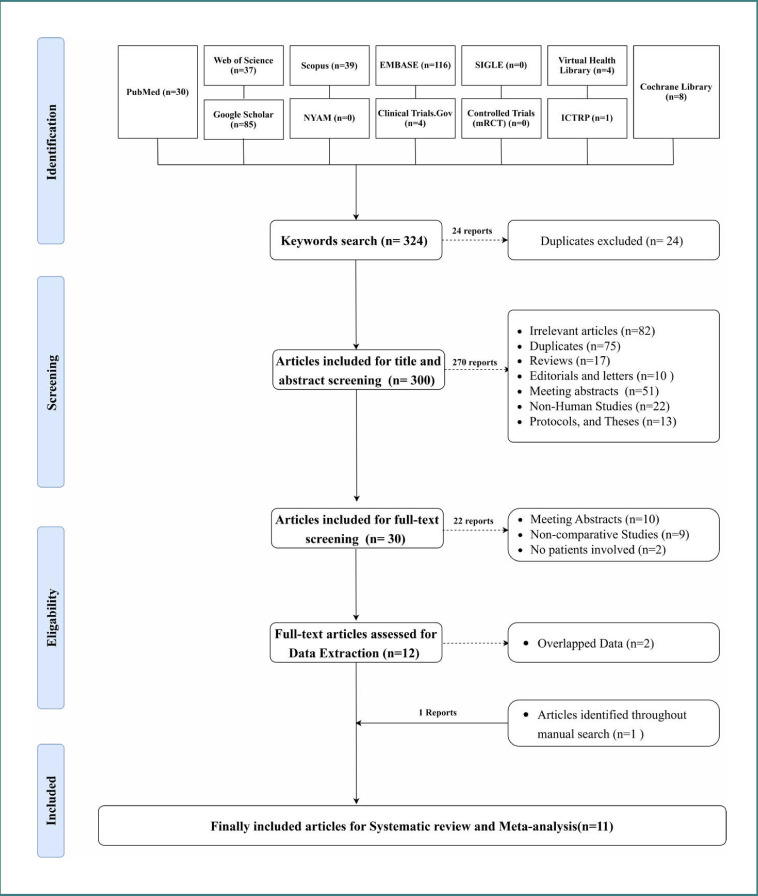
The PRISMA flowchart of this study

A comprehensive search of the available literature turned up a total of 324 different papers. Following the title and abstract screening process, 300 research papers were identified as eligible, and 24 were excluded. The preliminary review identified thirty publications that should proceed to the full-text screening phase. There were 14 publications considered for inclusion in the data extraction process, but only 12 were used. Throughout the manual search, one article was located, which led to the discovery of eleven publications suitable for systematic review and meta-analysis. The search strategies employed for the databases are outlined in Supplementary Table 2, and the screening procedures are illustrated in the PRISMA flowchart (Figure 1).

Supplementary Table 2

### Demographic characteristics

The present meta-analysis included eleven articles, encompassing 759 patients with CKD [25-35]. Of them, 381 patients were in the mobile applications group, while 378 patients were in the control group. Five of the included articles were designed as randomized controlled trials (RCTs), while the rest followed an observational design. The average age of the included patients ranged from 43 to 64.7 years. There were 221 females and 58 non-black patients. There were 87 patients with a high-school education, and 76 had a college degree. Of the included patients, 87 and 12 had full-time and part-time employment statuses, respectively (Table 1).

**Table 1:** Demographic characteristics of the included studies

Study ID		Study Region	Study Design	Registration Number	Study Period									Education						Employment status			
						Sample Size		Age (Years)		Gender (Females)		Race (Non-Black)		High school		Some college		College degree		Full Time		Part-Time	
						I	C	I	C	I	C	I	C	I	C	I	C	I	I	I	C	I	C
						n	n	Mean± SD	Mean± SD	n	n	n	n	n	n	n	n	n	n	n	n	n	n
1	Chang *et al*., 2020	USA	a pre-post, mixed methods	NA	April- November 2016	16	16	64.7 (48-86)*		5		16		6		5		5		6		3	
2	Diamantidis *et al*., 2015	USA	RCT	NCT 01407367	January and September of 2013	10	10	NR	NR	3	5	3	2	NR	NR	4	2	6	8	NR	NR	NR	NR
3	Ellis *et al*., 2019	USA	longitudinal study	NA	March 2018 and June 2018	5	5	52.6±22.49		2		4		2		1		1		2		NR	NR
4	Han *et al*., 2019	Korea	RCT	NCT 01905514	NR	70	66	45 (35–54)*	43 (30–52)*	27	21	NR	NR	27	21	30	32	NR	NR	37	30	4	5
5	Hayashi *et al*., 2017	Japan	Pilot Study	NA	NR	9	11	47.9± 14.4	60.3± 10.5	3	4	NR	NR	NR	NR	NR	NR	NR	NR	NR	NR	NR	NR
6	Imtiaz *et al*., 2017	Canada	Pilot Study	NA	March 2016 to July 2016	10	10	55.4 (17.5)		5		NR	NR	1		2		3		2		NR	NR
7	Li *et al*., 2020	Taiwan	RCT	NCT04617431	NR	25	24	50.60 ± 11.87	51.87± 10.20	8	6	NR	NR	5	13	NR	NR	20	9	NR	NR	NR	NR
8	Maneesri *et al*., 2023	Thailand.	RCT	NR	NR	20	20	NR	NR	5	7	NR	NR	NR	NR	NR	NR	NR	NR	NR	NR	NR	NR
9	Ong *et al*., 2016	Canada	Pilot Study	NA	NR	57	57	59.4±14		31		33		12		NR	NR	24		NR	NR	NR	NR
10	Pinto *et al*., 2019	Brazil	RCT	NR	January 2018 to April 2018	52	52	NR	NR	NR	NR	NR	NR	NR	NR	NR	NR	NR	NR	NR	NR	NR	NR
11	Tsai *et al*., 2021	Taiwan	Cross-sectional survey	NA	NR	107	107	63.5 ± 11.1	64.2 ± 11.9	40	47	NR	NR	NR	NR	NR	NR	NR	NR	NR	NR	NR	NR

RCT=Randomized Controlled Trial, NA=Non-applicable, NR=Non-reported, * Median and Range, I=Intervention, C=Control

There were 315 patients with hypertension. Diabetes mellitus was encountered among 136 patients, whereas cardiovascular diseases and dyslipidemia were revealed among 50 and 12 patients. The average baseline glumerular filtration rate (GFR) ranged from 64.27 to 76.2 ml/min/1.73m2 (Table 2).

**Table 2:** Comprehensive Summaries of the Included Studies

Study ID		Comorbidities										eGFR, ml/min/1.73m2		Comparative arm	Follow-up Period	Quality Assessment	
		Hypertension		Diabetes Mellitus		CVD		Dyslipidemia		Current Smokers							
		I	C	I	C	I	C	I	C	I	C	I	C				
		n	n	n	n	n	n	n	n	n	n	Mean ± SD	Mean ± SD			%	Decision
1	Chang *et al*., 2020	13		11		NR	NR	10		1		76.2± 16		Baseline	Eight Weeks	84.6%	Good
2	Diamantidis *et al*., 2015	NR	NR	8	6	6	7	NR	NR	NR	NR	NR	NR	short messaging service text	90 Days	______	______
3	Ellis *et al*., 2019	5		1		3		NR	NR	NR	NR	NR	NR	Baseline	25 Days	61.5%	Good
4	Han *et al*., 2019	5	3	4	4	NR	NR	NR	NR	1	3	NR	NR	conventional care	6-month intervention period	______	______
5	Hayashi *et al*., 2017	8	7	2	3	1	1	1	1	NR		NR	NR	Non-Smart Group	NR	76.92%	Good
6	Imtiaz *et al*., 2017	NR	NR	NR	NR	NR	NR	NR	NR	NR	NR	NR	NR	Baseline	NR	66.66%	Fair
7	Li *et al*., 2020	11	12	8	9	16	16	NR	NR	NR	NR	73.03± 25.01	64.27± 22.72	conventional care	90 Days	______	______
8	Maneesri *et al*., 2023	18	18	NR	NR	NR	NR	NR	NR	NR	NR	NR	NR	Educational Program Only	NR	______	______
9	Ong *et al*., 2016	5		7		NR	NR	NR	NR	NR	NR	NR	NR	NR	6 months	76.92%	Good
10	Pinto *et al*., 2019	31		1		NR	NR	NR	NR	NR	NR	NR	NR	NR	four months	______	______
11	Tsai *et al*., 2021	90	89	38	34	NR	NR	NR	NR	28	27	NR	NR	Baseline	NR	76.92%	Good

CVD=Cardiovascular diseases, eGFR=Estimated Glomerular Filtration Rate, NR=Non-reported, I=Intervention, C=Control

### Risk of bias and quality assessment

**Figure 2:**
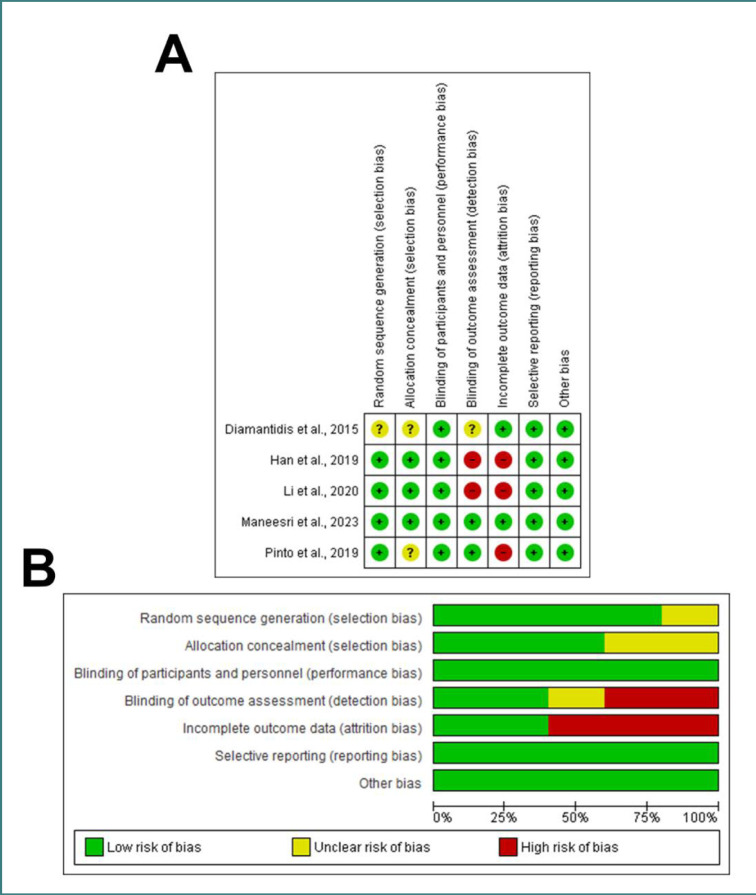
Risk of Bias and Quality Assessment

The potential for bias in the included RCTs was assessed. All of the included investigations, except for Diamantidis *et al*., 2015 [26], demonstrated a low risk of bias in producing random sequences [28, 31, 32, 34]. All of the publications [26, 28, 31, 32, 34] demonstrated a low risk of performance bias, and three studies [31, 32, 34] found a low risk of allocation concealment bias. There was a high risk of attribution bias in three papers [28, 31, 34] and a high risk of detection bias in two articles [28, 31]. The selected observational studies all showed a high quality [25, 29, 30, 33, 35], except for Ellis *et al*. [27], which only demonstrated a moderate quality (Figure 2 A-B and Table 2, respectively).

### Study endpoints

#### Glomerular filtration

**Figure 3:**
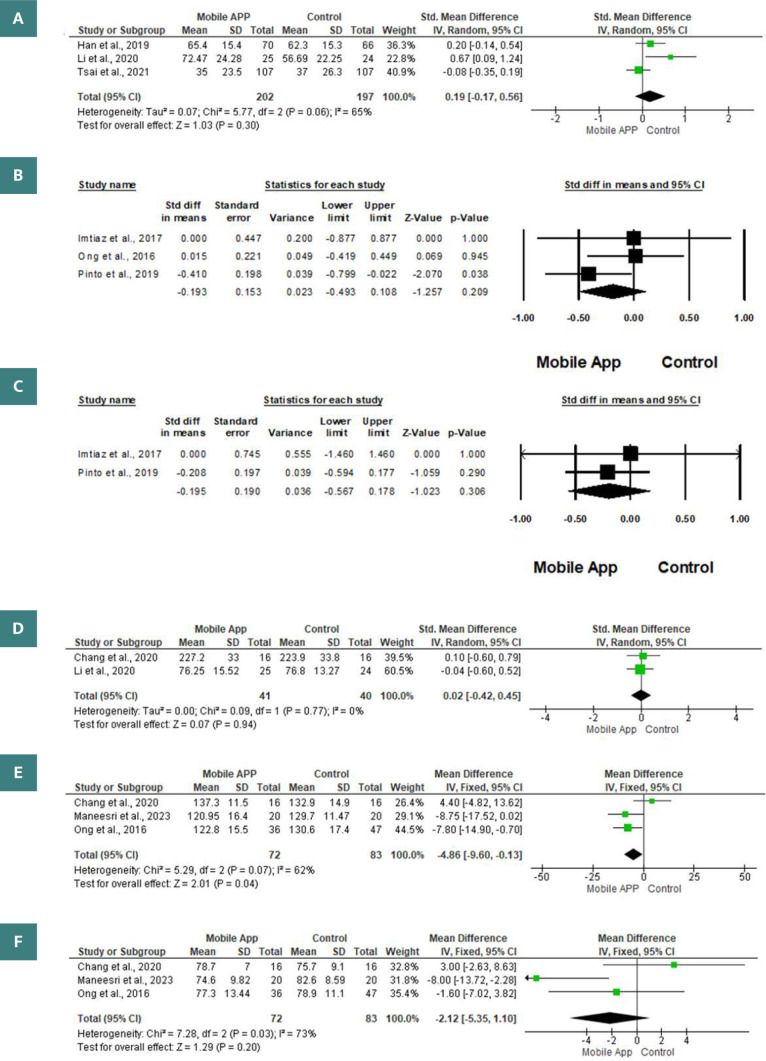
Study endpoints, including (A) glomerular filtration rate, (B) serum phosphate, (C) serum calcium, (D) body weight, (E) systolic blood pressure, and (F) diastolic blood pressure

Three articles included 399 patients with CKD and evaluated the impact of mobile health applications on the mean values of glomerular filtration rate. Using the random-effects model (I2=65%, p=0.06), the study found that there was no significant difference between the groups that used the mobile application and those that did not (SMD 0.19; 95% -0.17, 0.56; p=0.30) (Figure 3A).

#### Serum phosphate

Three studies included 207 patients and assessed the mean serum phosphate levels difference between the mobile application and control groups [30, 33, 34]. Based on a random-effects model (I2=1.82%, p=0.32), there was no significant difference between the mobile app and control groups (SMD -0.193; 95% -0.493, 0.108; p=0.209) (Figure 3B).

#### Serum calcium

The difference in mean serum calcium levels between the mobile application and control groups was revealed in two articles following 62 patients with CKD [30, 34]. When comparing patients who used the mobile app to those who did not, the random-effects model found no difference (SMD -0.193; 95% -0.493, 0.108; p=0.209) (Figure 3C).

#### Body weight

The difference in body weight between the mobile application and control group among patients with CKD was reported in two articles, including 81 patients [25, 31]. There was no statistically significant difference between the two groups according to the random-effects model (I2=0%, p=0.77) (SMD=0.02; 95% -0.42, 0.45; p=0.94) (Figure 3D).

#### Blood pressure

The influence of mobile applications on the average levels of systolic blood pressure (SBP) and diastolic blood pressure (DBP) in 155 patients with chronic kidney disease was evaluated in three articles [25, 32, 33]. There was a statistically significantly lower mean SBP (MD -4.86; 95% -9.60, -0.13; p=0.04) among patients in the mobile application group relative to the control group. Furthermore, there was not a statistically significant difference between either group in terms of the mean levels of DBP (median difference: -2.12; 95% confidence interval: -5.35, 1.10; p=0.20) (Figures 3E and 3F).

#### Medication adherence

**Figure 4:**
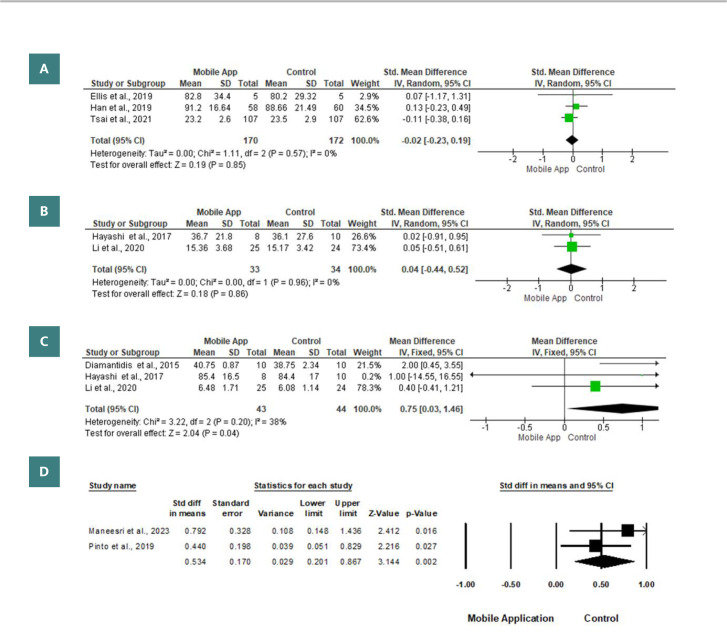
Study endpoints, including (A) medication adherence, (B) the burden of kidney disease, (C) patient satisfaction, and (D) self-management

The difference in medication adherence between the mobile application and the control groups was evaluated in three studies involving a total of 342 patients diagnosed with CKD [27, 28, 35]. The random-effects model showed no significant difference between the two groups (standard mean difference=-0.02; 95% confidence interval=-0.23, 0.19; p=0.85) (Figure 4A).

#### The burden of kidney disease

The effects of mobile applications on the burden of chronic renal disease were investigated in two trials with 67 patients diagnosed with CKD [29, 31]. The random-effects model showed that there was no significant difference between the mobile application group and the control group (SMD 0.04; 95% -0.44, 0.52; p=0.86) There was no significant difference between the mobile application group and the control group (Figure 4B).

#### Patient satisfaction

Three studies included 87 patients with CKD and evaluated the mean levels of patients’ satisfaction between mobile applications and control groups [26, 29, 31]. Patients in the mobile application group revealed a statistically significantly higher mean level of satisfaction, in contrast to patients in the control group (MD 0.75; 95% 0.03, 1.46; p=0.04) in the fixed-effect model (I2=38%, p=0.20) (Figure 4C).

### Self-management

In two separate studies [32, 34], 144 CKD patients were included in an analysis comparing self-management levels between those using the mobile application and the control group. When the data were combined using the random-effects model (I2=38%, p=0.20), it was revealed that the mobile application group had significantly higher mean levels of self-management (SMD 0.534; 95% CI 0.201, 0.867; p=0.002) compared to the fixed-effect model (I2=0%, p=0.358) (Figure 4D).

## DISCUSSION

Over the past century, there has been a significant transformation in the delivery of care for individuals with CKD. The traditional provider-centered approach has been replaced by a patient-centered framework, leading to increased patient engagement in the decision-making process and the evolution of technologies to support the self-management of CKD [36]. The adverse events associated with uncontrolled CKD underscore the necessity for customized digital tools to safeguard patients [37]. The usability and effectiveness of mobile applications have been revealed for different chronic diseases [38-40]. However, the available literature regarding the efficacy and usability of mobile applications for managing patients with CKD needs to be more conclusive. As a result, this meta-analysis was carried out to draw definitive evidence from the existing body of research about the impact that mobile health applications have on CKD outcomes. The present meta-analysis revealed the acceptability and feasibility of mobile health applications for managing patients with CKD. Mobile applications considerably improved the self-management of CKD with favorable SBP and patient satisfaction outcomes. However, mobile health applications had no significant impact on renal function tests, medication adherence, and the burden of kidney disease.

Mobile applications offer an effective means of providing care to patients with CKD. In line with this finding, Stahr *et al*., 2022, revealed that smartphone applications improve the understanding of chronic disorders with significant enhancement of self-management [41]. Mobile health applications offer an individualized system for patients with chronic diseases. The applications provide measures to increase the knowledge and understanding of the disease progression and opportunities for effective and timely management [42]. Contrary to these findings, Virella Pérez *et al*., revealed limited effectiveness of mhealth apps for managing adults with chronic disorders [43]. The effectiveness of mhealth apps is attributable to many factors. This included easy integration into daily life, tailoring the applications to the target patients, and adequate training of patients. The educational programs provided by the applications improve the relationship between the patients and the disease, increasing the effectiveness of mobile health educational programs [9, 44, 45].

In the present meta-analysis, mobile health applications had a significant impact on SBP. In this respect, Li *et al*. revealed the efficacy of mobile health applications for controlling blood pressure. This was accomplished with significant improvement in self-management and medication adherence [46]. Khoong *et al*. (2021) revealed the effectiveness of mobile technology in managing elevated blood pressure among the vulnerable population [47].

Dietary mobile health applications can be used as a complement to nutrition screening practice. This technology can help dietitians mitigate nutritional problems in patients with chronic diseases. This reduces the reliance on traditional dietary control methods [48]. Paradoxically, the present meta-analysis revealed non-significant changes in nutrient intake, including serum calcium and phosphate. Furthermore, mobile health applications had no significant impact on renal functions, body weight, and the burden of kidney disease. These findings were consistent with Campbell *et al*. [49]. They reported that dietary mobile applications have no substantial effects on biochemical parameters, nutrient intake, or weight gain in patients with CK. Moreover, Russell *et al*. [50] reported that none of the available dietary applications were based on nutritional guidelines for CKD management. The shortcomings of these applications included the requirement of a high educational level, lack of privacy, insecurity, limited interactive features, barriers to usability, and inaccurate information. These issues can lead to increased patient involvement in managing their illness, which can amplify the perceived severity of the disease and negatively impact their quality of life [51, 52]. New strategies are needed to enhance the engagement of patients with CKD in healthcare mobile application systems.

This meta-analysis collected contentious information addressing the influence that mobile health applications have on the outcomes of CKD patients. On the other hand, while evaluating the results of the investigation, some caveats need to be taken into account. Although five randomized controlled trials were included in this meta-analysis, most of the studies used observational methods. This demonstrated that there was a potential danger of selection bias. Furthermore, there was noticeable heterogeneity among the studies, likely due to variations in study design, mobile applications used, demographic factors, and research outcomes. To address these shortcomings, future research should involve more RCTs with larger sample sizes and longer follow-up periods.

## CONCLUSION

Mobile health applications are potentially valuable interventions for patients with chronic kidney disease. This technology improved the self-management of the disease, reducing the mean levels of systolic blood pressure with a high degree of patient satisfaction. Implementing mobile health applications improves healthcare quality for patients with chronic kidney disease by providing an accessible, feasible, and effective self-management intervention.

## Data Availability

All data generated or analyzed during this study are included in this published article (and its supplementary information files).

## Personal thanks

The authors would like to express their deepest gratitude to the UGM Directorate of Research and UGM Reputation Improvement Team towards World Class University and the Directorate of Research.

## References

[1] Lopez-Vargas PA, Tong A, Howell M, Phoon RK, Chadban SJ, Chadban SJ (2017). Patient awareness and beliefs about the risk factors and comorbidities associated with chronic kidney disease: A mixed-methods study. Nephrology (Carlton).

[2] Woo KT, Choong HL, Wong KS, Tan HB, Chan CM (2012). The contribution of chronic kidney disease to the global burden of major noncommunicable diseases. Kidney Int.

[3] Kumela Goro K, Desalegn Wolide A, Kerga Dibaba F, Gashe Fufa F (2019). Patient Awareness, Prevalence, and Risk Factors of Chronic Kidney Disease among Diabetes Mellitus and Hypertensive Patients at Jimma University Medical Center, Ethiopia. Biomed Res Int.

[4] Kovesdy CP (2022). Epidemiology of chronic kidney disease: an update 2022. Kidney Int Suppl (2011).

[5] Norton JM, Moxey-Mims MM, Eggers PW, Narva AS (2016). Social Determinants of Racial Disparities in CKD. J Am Soc Nephrol.

[6] Chen SH, Tsai YF, Sun CY, Wu IW (2011). The impact of self-management support on the progression of chronic kidney disease--a prospective randomized controlled trial. Nephrol Dial Transplant.

[7] Younes S, Mourad N, Safwan J, Dabbous M (2022). Chronic kidney disease awareness among the general population: tool validation and knowledge assessment in a developing country. BMC Nephrology.

[8] Yang Y, Chen H, Qazi H, Morita PP (2020). Intervention and Evaluation of Mobile Health Technologies in Management of Patients Undergoing Chronic Dialysis: Scoping Review. JMIR Mhealth Uhealth.

[9] Free C, Phillips G, Watson L, Galli L (2013). The effectiveness of mobile-health technologies to improve health care service delivery processes: a systematic review and meta-analysis. PLoS Med.

[10] Sobrinho A, da Silva LD, Perkusich A, Pinheiro ME, Cunha P (2018). Design and evaluation of a mobile application to assist the self-monitoring of the chronic kidney disease in developing countries. BMC Med Inform Decis Mak.

[11] Mashkoor A (2016). Model-driven development of high-assurance active medical devices. Soft Qual J.

[12] Control C for D Prevention (2019). Chronic kidney disease in the United States, 2019. Atlanta, GA: US Department of Health and Human Services, Centers for Disease Control and Prevention.

[13] Burke MT, Kapojos J, Sammartino C, Gray NA (2014). Kidney disease health literacy among new patients referred to a nephrology outpatient clinic. Intern Med J.

[14] Gray NA, Kapojos JJ, Burke MT, Sammartino C, Clark CJ (2016). Patient kidney disease knowledge remains inadequate with standard nephrology outpatient care. Clin Kidney J.

[15] Ghozali MT (2022). Mobile app for COVID-19 patient education–Development process using the analysis, design, development, implementation, and evaluation models: Nonlinear Eng.

[16] Sadler E, Wolfe CDA, McKevitt C (2014). Lay and health care professional understandings of self-management: A systematic review and narrative synthesis. SAGE Open Med.

[17] Moher D, Liberati A, Tetzlaff J, Altman DG, PRISMA Group (2009). Preferred reporting items for systematic reviews and meta-analyses: the PRISMA statement. BMJ.

[18] Higgins JP, Green S, Collaboration C (2008). Cochrane handbook for systematic reviews of interventions. Chichester.

[19] Higgins JPT, Altman DG, Gøtzsche PC, Jüni P (2011). The Cochrane Collaboration’s tool for assessing risk of bias in randomised trials. BMJ.

[20] National Heart Institute (2014). Quality assessment tool for observational cohort and cross-sectional studies.

[21] Hozo SP, Djulbegovic B, Hozo I (2005). Estimating the mean and variance from the median, range, and the size of a sample. BMC Med Res Methodol.

[22] Higgins JPT, Thompson SG, Deeks JJ, Altman DG (2003). Measuring inconsistency in meta-analyses. BMJ.

[23] Borenstein M, Hedges L, Higgins JPT, Rothstein HR (2005). Comprehensive meta-analysis (Version 2.2027) [Computer software].

[24] Collaboration C (2014). Review manager (version 5.3)[computer software]. Copenhagen Denmark Nord Cochrane Centre, Cochrane Collab.

[25] Chang AR, Bailey-Davis L, Hetherington V, Ziegler A (2020). Remote Dietary Counseling Using Smartphone Applications in Patients With Stages 1-3a Chronic Kidney Disease: A Mixed Methods Feasibility Study. J Ren Nutr.

[26] Diamantidis CJ, Ginsberg JS, Yoffe M, Lucas L (2015). Remote Usability Testing and Satisfaction with a Mobile Health Medication Inquiry System in CKD. Clin J Am Soc Nephrol.

[27] Bartlett Ellis RJ, Hill JH, Kerley KD, Sinha A (2019). The Feasibility of a Using a Smart Button Mobile Health System to Self-Track Medication Adherence and Deliver Tailored Short Message Service Text Message Feedback (Preprint). JMIR Formative Research.

[28] Han A, Min S il, Ahn S, Min SK (2019). Mobile medication manager application to improve adherence with immunosuppressive therapy in renal transplant recipients: A randomized controlled trial. PLoS One.

[29] Hayashi A, Yamaguchi S, Waki K, Fujiu K (2017). Testing the Feasibility and Usability of a Novel Smartphone-Based Self-Management Support System for Dialysis Patients: A Pilot Study. JMIR Res Protoc.

[30] Imtiaz R, Atkinson K, Guerinet J, Wilson K (2017). A Pilot Study of Okkidney, A Phosphate Counting Application in Patients on Peritoneal Dialysis. Perit Dial Int.

[31] Li WY, Chiu FC, Zeng JK, Li YW (2020). Mobile Health App With Social Media to Support Self-Management for Patients With Chronic Kidney Disease: Prospective Randomized Controlled Study. J Med Internet Res.

[32] Maneesri S, Masingboon K, Chaimongkol N (2023). Effectiveness of Individual and Family Self-Management Combined mHealth Program for People with Stage 3 Chronic Kidney Disease: A Randomized Controlled Trial. Pacific Rim International Journal of Nursing Research.

[33] Ong SW, Jassal SV, Miller JA, Porter EC (2016). Integrating a Smartphone–Based Self–Management System into Usual Care of Advanced CKD. CJASN.

[34] Pinto LCS, Andrade MC, Chaves RO, Lopes LLB (2020). Development and Validation of an Application for Follow-up of Patients Undergoing Dialysis: NefroPortátil. J Ren Nutr.

[35] Tsai YC, Hsiao PN, Kuo MC, Wang SL (2021). Mobile Health, Disease Knowledge, and Self-Care Behavior in Chronic Kidney Disease: A Prospective Cohort Study. JPM.

[36] Schell JO, Arnold RM (2012). NephroTalk: communication tools to enhance patient-centered care. Semin Dial.

[37] Wright Nunes JA, Wallston KA, Eden SK, Shintani AK (2011). Associations among perceived and objective disease knowledge and satisfaction with physician communication in patients with chronic kidney disease. Kidney Int.

[38] Agarwal P, Gordon D, Griffith J, Kithulegoda N (2021). Assessing the quality of mobile applications in chronic disease management: a scoping review. NPJ Digit Med.

[39] Osei E, Mashamba-Thompson TP (2021). Mobile health applications for disease screening and treatment support in low-and middle-income countries: A narrative review. Heliyon.

[40] Iribarren SJ, Akande TO, Kamp KJ, Barry D (2021). Effectiveness of Mobile Apps to Promote Health and Manage Disease: Systematic Review and Meta-analysis of Randomized Controlled Trials. JMIR Mhealth Uhealth.

[41] Stahr B, Fudickar SJ, Lins C (2022). Mobile Applications for Self-management of Chronic Diseases: A Systematic Review. HEALTHINF.

[42] Schnall R, Cho H, Mangone A, Pichon A, Jia H (2018). Mobile Health Technology for Improving Symptom Management in Low Income Persons Living with HIV. AIDS Behav.

[43] Virella Pérez YI, Medlow S, Ho J, Steinbeck K (2019). Mobile and Web-Based Apps That Support Self-Management and Transition in Young People With Chronic Illness: Systematic Review. J Med Internet Res.

[44] Free C, Phillips G, Galli L, Watson L, Cornford T (2013). The Effectiveness of Mobile-Health Technology-Based Health Behaviour Change or Disease Management Interventions for Health Care Consumers: A Systematic Review. PLoS Med.

[45] Balapour A, Reychav I, Sabherwal R, Azuri J (2019). Mobile technology identity and self-efficacy: Implications for the adoption of clinically supported mobile health apps. Int J Inf Manage.

[46] Li R, Liang N, Bu F, Hesketh T (2020). The Effectiveness of Self-Management of Hypertension in Adults Using Mobile Health: Systematic Review and Meta-Analysis. JMIR Mhealth Uhealth.

[47] Khoong EC, Olazo K, Rivadeneira NA, Thatipelli S (2021). Mobile health strategies for blood pressure self-management in urban populations with digital barriers: systematic review and meta-analyses. npj Digit Med.

[48] Fakih El Khoury C, Karavetian M, Halfens RJG, Crutzen R (2019). The Effects of Dietary Mobile Apps on Nutritional Outcomes in Adults with Chronic Diseases: A Systematic Review and Meta-Analysis. J Acad Nutr Diet.

[49] Campbell J, Porter J (2015). Dietary mobile apps and their effect on nutritional indicators in chronic renal disease: A systematic review: Dietary apps in chronic renal disease. Nephrology.

[50] Russell CR, Zigan C, Wozniak K, Soni K (2022). A Systematic Review and Qualitative Analysis of Existing Dietary Mobile Applications for People With Chronic Kidney Disease. Journal of Renal Nutrition.

[51] Biduski D, Bellei EA, Rodriguez JPM, Zaina LAM, De Marchi ACB (2020). Assessing long-term user experience on a mobile health application through an in-app embedded conversation-based questionnaire. Comput Human Behav.

[52] Amagai S, Pila S, Kaat AJ, Nowinski CJ, Gershon RC (2022). Challenges in Participant Engagement and Retention Using Mobile Health Apps: Literature Review. J Med Internet Res.

