# Supplementary material for: The impact of mobile health applications on the outcomes of patients with chronic kidney disease: a systematic review and meta-analysis
Source: J Med Life. 2023 Sep;16(9):1299–309. doi: 10.25122/jml-2023-0153 (PMC10719786; doi:10.25122/jml-2023-0153)
Supplement: Supplementary file 1 [file JMedLife-16-1299-s001.pdf]

Supplementary Material

Supplementary Table 1. PRISMA 2009. Checklist [17]

Section/topic	#	Checklist item	Reported on page #
**TITLE**			
Title	1	Identify the report as a systematic review, meta-analysis, or both.	1
**ABSTRACT**			
Structured summary	2	Provide a structured summary including, as applicable: background; objectives; data sources; study eligibility criteria, participants, and interventions; study appraisal and synthesis methods; results; limitations; conclusions and implications of key findings; systematic review registration number.	2
**INTRODUCTION**			
Rationale	3	Describe the rationale for the review in the context of what is already known.	3-4
Objectives	4	Provide an explicit statement of questions being addressed with reference to participants, interventions, comparisons, outcomes, and study design (PICOS).	4
**METHODS**			
Protocol and registration	5	Indicate if a review protocol exists, if and where it can be accessed (e.g., Web address), and, if available, provide registration information, including registration number.	4
Eligibility criteria	6	Specify study characteristics (e.g., PICOS, length of follow-up) and report characteristics (e.g., years considered, language, publication status) used as criteria for eligibility, giving rationale.	5
Information sources	7	Describe all information sources (e.g., databases with coverage dates, contact with study authors to identify additional studies) in the search and the date last searched.	5
Search	8	Present a full electronic search strategy for at least one database, including any limits used, such that it could be repeated.	5
Study selection	9	State the process for selecting studies (i.e., screening, eligibility, included in the systematic review, and, if applicable, included in the meta-analysis).	5-6
Data collection process	10	Describe method of data extraction from reports (e.g., piloted forms, independently, in duplicate) and any processes for obtaining and confirming data from investigators.	6
Data items	11	List and define all variables for which data were sought (e.g., PICOS, funding sources) and any assumptions and simplifications made.	6
Risk of bias in individual studies	12	Describe methods used for assessing risk of bias in individual studies (including specification of whether this was done at the study or outcome level) and how this information is to be used in any data synthesis.	5
Summary measures	13	State the principal summary measures (e.g., risk ratio, difference in means).	7
Synthesis of results	14	Describe the methods of handling data and combining results of studies, if done, including measures of consistency (e.g., I^2) for each meta-analysis.	6-7
Risk of bias across studies	15	Specify any assessment of risk of bias that may affect the cumulative evidence (e.g., publication bias, selective reporting within studies).	6
Additional analyses	16	Describe methods of additional analyses (e.g., sensitivity or subgroup analyses, meta-regression), if done, indicating which were pre-specified.	NA
**RESULTS**			
Study selection	17	Give the number of studies screened, assessed for eligibility, and included in the review, with reasons for exclusions at each stage, ideally with a flow diagram.	7
Study characteristics	18	For each study, present characteristics for which data were extracted (e.g., study size, PICOS, follow-up period) and provide the citations.	7
Risk of bias within studies	19	Present data on risk of bias of each study and, if available, any outcome level assessment (see item 12).	8 and Figure.2
Results of individual studies	20	For all outcomes considered (benefits or harms), present for each study: (a) simple summary data for each intervention group, (b) effect estimates and confidence intervals, ideally with a forest plot.	8-10 AND Tables 1 and 2

Licensed under CC BY 4.0

Supplementary Table 1. Continued. PRISMA 2009. Checklist

Section/topic	#	Checklist item	Reported on page #
Synthesis of results	21	Present results of each meta-analysis done, including confidence intervals and measures of consistency.	8-10 and Figures 3 and 4
Risk of bias across studies	22	Present results of any assessment of risk of bias across studies (see Item 15).	8 Figure.2
Additional analysis	23	Give results of additional analyses, if done (e.g., sensitivity or subgroup analyses, meta-regression [see Item 16]).	NA
**DISCUSSION**			
Summary of evidence	24	Summarize the main findings, including the strength of evidence for each main outcome; consider their relevance to key groups (e.g., healthcare providers, users, and policymakers).	10-11
Limitations	25	Discuss limitations at the study and outcome levels (e.g., risk of bias) and review levels (e.g., incomplete retrieval of identified research, reporting bias).	12
Conclusions	26	Provide a general interpretation of the results in the context of other evidence and implications for future research.	13
**FUNDING**			
Funding	27	Describe sources of funding for the systematic review and other support (e.g., supply of data); role of funders for the systematic review.	Title Page

Licensed under CC BY 4.0

Supplementary Material

Supplementary Table 2

	Database	Date	Keywords	Results	Link
1	PubMed	1-3-2023	((((((Mobile application[Title]) OR (Mobile app[Title])) OR (m health[Title])) OR (mhealth[Title])) OR (smart phone[Title])) OR (smartphone[Title])) OR (tablet[Title])) AND (Chronic Kidney[Title])	16	Link
			((((((Mobile application[Title]) OR (Mobile app[Title])) OR (m health[Title])) OR (mhealth[Title])) OR (smart phone[Title])) OR (smartphone[Title])) OR (tablet[Title])) AND (Chronic Renal[Title])	3	Link
			((((((Mobile application[Title]) OR (Mobile app[Title])) OR (m health[Title])) OR (mhealth[Title])) OR (smart phone[Title])) OR (smartphone[Title])) OR (tablet[Title])) AND (CKD[Title])	6	Link
			((((((Mobile application[Title]) OR (Mobile app[Title])) OR (m health[Title])) OR (mhealth[Title])) OR (smart phone[Title])) OR (smartphone[Title])) OR (tablet[Title])) AND (Dialysis[Title])	5	Link
2	Google Scholar	1-3-2023	allintitle: Chronic Kidney "Mobile application" OR "Mobile app" OR "m health" OR mhealth OR "smart phone" OR smartphone OR tablet	41	Link
			allintitle: Chronic renal "Mobile application" OR "Mobile app" OR "m health" OR mhealth OR "smart phone" OR smartphone OR tablet	16	Link
			allintitle: CKD "Mobile application" OR "Mobile app" OR "m health" OR mhealth OR "smart phone" OR smartphone OR tablet	11	Link
			allintitle: Dialysis "Mobile application" OR "Mobile app" OR "m health" OR mhealth OR "smart phone" OR smartphone OR tablet	17	Link
3	Web of Science	1-3-2023	TITLE: (Mobile application) OR TITLE: (Mobile app) OR TITLE: (m health) OR TITLE: (mhealth OR TITLE: (smart phone) OR TITLE: (smartphone) AND TITLE: (Chronic Kidney) OR TITLE: (Chronic Renal) OR TITLE: (CRD) OR TITLE: (Dialysis)	37	Link
4	Scopus	1-3-2023	(TITLE (mobile AND application) OR TITLE (mobile AND app) OR TITLE (m AND health) OR TITLE (mhealth) OR TITLE (smart AND phone) OR TITLE (smartphone) OR TITLE (tablet) AND TITLE (chronic AND kidney) OR SRCTITLE (chronic AND renal) OR TITLE (crd) OR TITLE (crf) OR TITLE (dialysis))	52	Link
5	EMBASE	1-3-2023	('mobile application'/exp OR 'mobile application' OR (mobile AND ('application'/exp OR application)) OR 'mobile app':ti OR 'm health':ti OR mhealth:ti OR 'smart phone':ti OR smartphone:ti OR tablet:ti) AND 'chronic kidney':ti	47	Link
			('mobile application'/exp OR 'mobile application' OR (mobile AND ('application'/exp OR application)) OR 'mobile app':ti OR 'm health':ti OR mhealth:ti OR 'smart phone':ti OR smartphone:ti OR tablet:ti) AND 'chronic renal':ti	8	Link
			('mobile application'/exp OR 'mobile application' OR (mobile AND ('application'/exp OR application)) OR 'mobile app':ti OR 'm health':ti OR mhealth:ti OR 'smart phone':ti OR smartphone:ti OR tablet:ti) AND 'ckd':ti	33	Link
			('mobile application'/exp OR 'mobile application' OR (mobile AND ('application'/exp OR application)) OR 'mobile app':ti OR 'm health':ti OR mhealth:ti OR 'smart phone':ti OR smartphone:ti OR tablet:ti) AND 'dialysis':ti	28	Link
6	SIGLE	1-3-2023	Mobile OR m-health OR mhealth OR "smart phone" OR smartphone OR tablet AND Chronic OR Kidney OR renal OR CKD OR Dialysis	0	Link
7	Virtual Health Library	1-3-2023	(ti:(Mobile)) OR (ti:(m-health)) OR (ti:(mhealth)) OR (ti:(smart phone)) OR (ti:(smart-phone)) OR (ti:(tablet)) AND (ti:(Chronic Kidney))	4	Link
8	NYAM	1-3-2023	Mobile OR m-health OR mhealth OR "smart phone" OR smartphone OR tablet AND Chronic OR Kidney OR renal OR CKD OR Dialysis	0	Link
9	Clinical Trials.Gov	1-3-2023	Renal | Chronic Kidney | Mobile | Smart Phone	3	Link
10	Controlled Trials (mRCT)	1-3-2023	"(Interventions: Mobile AND Trial acronym: Kidney AND Condition: Renal)"	0	Link
11	ICTRP	1-3-2023	Renal and Mobile Application	1	Link
12	Cochrane Library	1-3-2023	Mobile Application in Record Title AND Kidney in Record Title - (Word variations have been searched)	8	Link

Licensed under CC BY 4.0
